# Associations of Peak-Width Skeletonized Mean Diffusivity and Post-Stroke Cognition

**DOI:** 10.3390/life12091362

**Published:** 2022-08-31

**Authors:** Angela C. C. Jochems, Susana Muñoz Maniega, Una Clancy, Daniela Jaime Garcia, Carmen Arteaga, Will Hewins, Rachel Penman, Olivia K. L. Hamilton, Agnieszka Czechoń, Ellen V. Backhouse, Michael J. Thrippleton, Michael S. Stringer, Mark. E. Bastin, Maria del C. Valdés Hernández, Stewart Wiseman, Francesca M. Chappell, Fergus N. Doubal, Joanna M. Wardlaw

**Affiliations:** 1 Centre for Clinical Brain Sciences and UK Dementia Research Institute, The University of Edinburgh, Edinburgh EH16 4SB, UK; 2 Edinburgh Imaging Facility, Royal Infirmary of Edinburgh, Edinburgh EH16 4TJ, UK

**Keywords:** ischemic stroke, small vessel disease, cognition, diffusion imaging, peak-width of skeletonized mean diffusivity

## Abstract

Post-stroke cognitive impairment is common and can have major impact on life after stroke. Peak-width of Skeletonized Mean Diffusivity (PSMD) is a diffusion imaging marker of white matter microstructure and is also associated with cognition. Here, we examined associations between PSMD and post-stroke global cognition in an ongoing study of mild ischemic stroke patients. We studied cross-sectional associations between PSMD and cognition at both 3-months (N = 229) and 1-year (N = 173) post-stroke, adjusted for premorbid IQ, sex, age, stroke severity and disability, as well as the association between baseline PSMD and 1-year cognition. At baseline, (mean age = 65.9 years (SD = 11.1); 34% female), lower Montreal Cognitive Assessment (MoCA) scores were associated with older age, lower premorbid IQ and higher stroke severity, but not with PSMD (β_standardized_ = −0.116, 95% CI −0.241, 0.009; *p* = 0.069). At 1-year, premorbid IQ, older age, higher stroke severity and higher PSMD (β_standardized_ = −0.301, 95% CI −0.434, −0.168; *p* < 0.001) were associated with lower MoCA. Higher baseline PSMD was associated with lower 1-year MoCA (β_standardized_ = −0.182, 95% CI −0.308, −0.056; *p* = 0.005). PSMD becomes more associated with global cognition at 1-year post-stroke, possibly once acute effects have settled. Additionally, PSMD in the subacute phase after a mild stroke could help predict long-term cognitive impairment.

## 1. Introduction

Cognitive impairment after stroke is common [1,2,3]. This post-stroke cognitive impairment is related to physical impairments and depression and is associated with disability [4]. Impaired cognitive functioning can restrict participation in life after stroke, whether that is socially, at work or in the community [5], which in turn can have a negative impact on the quality of life post-stroke [6]. While there are social and environmental factors that can impact participation, stroke-related impairments are the strongest predictor of poor post-stroke participation [5]. In the UK, 65% of stroke survivors report severe disability [7]. Stroke is also associated with gait [8] and motor impairments [9], and dementia [10,11]. Even after a non-disabling stroke that leaves mild residual physical deficits, cognition can be affected. However, this might be overlooked as cognitive symptoms can be mild or even incorrectly perceived as being normal.

Peak-width of skeletonized mean diffusivity (PSMD), derived from diffusion tensor imaging (DTI), allows the quantification of microstructural white matter changes, before the damage becomes visible on conventional brain magnetic resonance imaging (MRI), e.g., as white matter hyperintensities (WMH). Additionally, PSMD seems to be more sensitive to microstructural changes than more conventional DTI metrics such as fractional anisotropy (FA) and mean diffusivity (MD) [12]. While PSMD uses FA images and MD values, it focusses on the MD values in the skeleton of the main white matter tracts. This is thought to avoid possible contamination of cerebrospinal fluid [12]. By using histogram analysis of the peak width of the MD values in the skeleton it avoids bias about parts of the brain that may be affected and is sensitive to subtle and widespread pathology [12,13]. High PSMD values are thought to resemble microstructural damage. PSMD is a relatively new imaging marker associated with cerebral small vessel disease (SVD), which is one of the commonest causes of stroke, and related to an increased risk of stroke and dementia [14]. SVD imaging features, including WMH, lacunes, perivascular spaces and microbleeds are also each associated with increased risk of stroke and dementia [15] and cognitive impairment [16]. PSMD can capture changes of SVD over time [12,17] and has been cross-sectionally associated with clinical outcomes and symptoms such as functional outcome [18] and cognition, particularly processing speed, in a range of groups including healthy older people [12,19,20,21], in late life depression [22]; after acute stroke [18]; in sporadic and genetic SVDs [12], and in mild cognitive impairment, or dementia [21,23,24]. Undamaged white matter is important for good cognitive functioning and white matter damage, as measured with PSMD, relates to disruption of cognitive functioning.

While evidence of cross-sectional associations between PSMD and cognition is emerging, it has not been widely examined in patients following stroke or longitudinally. So far, stroke research suggests that increased PSMD might mediate the effect of older age on poorer functional outcomes after stroke, in addition to direct contribution to poor functional outcomes [18]. In stroke- and dementia-free community-dwelling older adults, PSMD is negatively associated with processing speed, and negatively associated with processing speed and memory three years later [20]. 

Previously we found that in people with stroke, the associations between WMH volume and global cognition are stronger at one year and three years after stroke, compared to the associations soon after the stroke [25]. As PSMD seems to be associated with more aspects of long-term cognition in older people [20], we hypothesize that PSMD might be more strongly associated with global cognition at 1 year after stroke than in the subacute phase. Therefore, we aim to examine the relationship between PSMD and global post-stroke cognition and determine whether associations between PSMD and cognition vary at different time-points after mild stroke.

## 2. Materials and Methods

### 2.1. Participants 

As part of an ongoing prospective cohort study, the Mild Stroke Study 3 [26], we recruited adult patients >18 years, who presented to Edinburgh and Lothian stroke services with an acute minor (≤2 modified Rankin scale [mRS] [27]) lacunar or non-lacunar ischemic stroke. The final stroke diagnosis is based on presenting symptoms and signs, supplemented by diagnostic brain MRI or CT. We excluded participants with MRI contraindications, severe cardiac or respiratory disease, and major neurological conditions. Participants had baseline assessments within three months post-stroke, including MRI, clinical and cognitive assessments. Participants repeated MRI, clinical and cognitive assessments one year after baseline. When participants were not able to attend the visit in person, data were gathered via telephone interview. All participants provided written informed consent. Patient recruitment commenced in August 2018 and ended in December 2021. As the follow-up visits are still ongoing, the smaller number of participants that have attended their follow-up visits are unselected. They represent the study population as a whole. The study was approved by Southeast Scotland Regional Ethics Committee, reference 18/SS/0044.

### 2.2. Clinical and Cognitive Assessments

We collected medical history provided by the participants. This is supplemented by hospital medical records, general practitioner correspondence and a structured interview at time of the first visit. As part of the clinical assessments, we recorded history of diabetes mellitus, hypertension, hypercholesterolemia, and atrial fibrillation and smoking. We assessed stroke severity (National Institutes of Health Stroke Scale [NIHSS]) [28] and disability after stroke (mRS) at all visits. Scores on the NIHSS range from 0 to 42, higher scores indicate greater stroke severity [28]. The mRS ranges from 0 (no symptoms) to 5 (severe disability) [27]. At baseline, the mRS for all participants was ≤2 due to inclusion criteria. 

As part of a neuropsychological test protocol, premorbid intelligence (IQ) was assessed by the National Adult Reading Test (NART) [29] at baseline. The NART assesses the pronunciation of 50 irregular English words which has proven to be related to general intelligence [30] and it is associated with educational attainment [31]. We used the NART to assess premorbid IQ because pronunciation of irregular words is well maintained in people with mild to moderate dementia [32] and post-stroke [31]. When administered, the number of errors is noted. More errors on the NART relate to lower premorbid IQ [31]. Subsequently, we subtracted the number of errors from the total number of words to get the number of correct answers. 

At both baseline and 1-year visit, global cognition was assessed with the Montreal Cognitive Assessment (MoCA) [33]. The MoCA is a brief cognitive screening test to assess mild cognitive impairment and it covers memory, visuospatial abilities, executive functioning, attention, language and orientation in time and place. The scores range from 0 to 30 and a score of ≤25 might indicate cognitive impairment [33]. Alternative versions of the MoCA were used at each study visit to minimize learning effects [34,35]. 

### 2.3. Image Acquisition

Full details of the brain imaging scanning protocol for the Mild Stroke Study 3 have been published previously [26]. Briefly, participants underwent brain MRI within three months post-stroke and they were invited to repeat MRI one year later, on the same 3T scanner (Siemens Prisma, Erlangen, Germany). We monitor the MRI scanner with a quality assurance program to check for any scanner performance issues and maintain consistent function and image quality. Images were acquired using a 32-channel head coil (Siemens Healthcare, Erlangen, Germany). MRI protocol included 3D T1-weighted, T2-weighted, fluid attenuated inversion recovery-weighted (FLAIR), susceptibility weighted (SWI) and multi-shell diffusion imaging. The acquisitions were repeated at one year follow-up, but with a single-shell DTI acquisition (isotropic voxel size of 2 mm, 11 volumes at b = 0 and 64 volumes at b = 1000 s/mm^2^). The DTI protocol was designed so the baseline multi-shell acquisition contained the follow-up single-shell acquisition, so an equivalent single-shell could be processed at both time points. 

### 2.4. Imaging Analysis

Diffusion data were processed using TractoR version 3.3.5 ‘dpreproc’ pipeline [36]. Briefly, DICOM data were converted to NIfTI-1 format using ‘divest’ [37], corrected for susceptibility and eddy current induced distortions using topup and eddy from FSL version 6.0.1 [38,39,40], and the brain was masked using FSL’s brain extraction tool [41]. At baseline we kept only the diffusion-weighted volumes equivalent to the follow-up single-shell acquisition and a self-diffusion tensor model was fitted in each brain voxel. Parametric maps of FA and MD were derived from its eigenvalues with TractoR’s ‘tensorfit’ using an iterative weighted least-squares approach [42].

**Figure 1:**
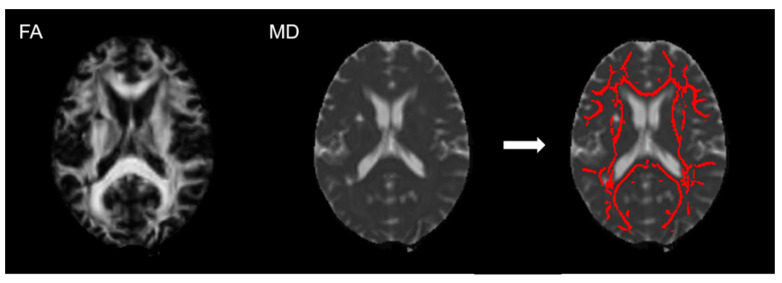
Example of application of peak-width of skeletonized mean diffusivity skeleton template. The fractional anisotropy (FA; **left**) images were normalized to a standard space and projected into a white matter skeleton template. This transformation was applied to the mean diffusivity (MD) images (**middle**) and MD skeleton was obtained (**right**).

We calculated PSMD using a previously described protocol [12] and the publicly available pipeline for fully processed DTI images (www.psmd-marker.com; accessed on 1 April 2022), which we adjusted to exclude acute stroke lesions. Briefly, the FA images were normalized to standard space and projected into a white matter skeleton template (Figure 1). The transformation was then applied to the MD maps and the projections used to obtain the MD skeleton. After histogram analysis, the peak width of the MD values within the skeleton was quantified. The peak width refers to the difference between the 5th and 95th percentile. Stroke areas were excluded from the skeleton to avoid effects of the lesion in PSMD values. For this, we used stroke masks manually drawn on the FLAIR volumes by an experienced rater, guided by the DWI data previously mapped in the structural space. All cases were discussed with a neuroradiologist and masks adjusted when required.

### 2.5. Statistical Analysis 

We ran three multivariable linear regressions. To assess cross-sectional relations, we ran models one linear regression model with MoCA at baseline as outcome, this model was adjusted for contemporaneous age, sex, premorbid IQ (NART), stroke severity (NIHSS), disability (mRS), and PSMD. The second cross-sectional linear regression model had the MoCA at 1 year as outcome. We adjusted the models for age, sex, NART, NIHSS, mRS and PSMD. Apart from, sex and NART, all variables were measured at 1-year visit. To explore associations between baseline PSMD and global cognition at 1 year, we ran a third linear regression model with 1-year MoCA as outcome adjusted for age, sex, premorbid IQ, NIHSS, mRS, MoCA, and PSMD. All the independent variables for the third linear regression were as gathered at baseline. In these analyses, we used the number of correct answers for the NART, thus higher NART scores relate to higher premorbid IQ. To avoid scaling problems, we multiplied PSMD by 1000 and converted to units of ×10^−4^ mm^2^/s. The models are not corrected for multiple comparisons in this explorative analysis. Model assumptions were met for all models. For the results, we have chosen to report standardized betas instead of the original units in order to be able to compare the influence of the variables, which would not be possible if we used the original units. All analyses were performed using R version 4.0.2 [43].

## 3. Results

### 3.1. Participants

An overview of the demographic, clinical, cognitive and PSMD data is shown in Table 1. The total number of participants at baseline was 229 (33.6% female), 226 provided MRI data that we could use to compute PSMD. The mean age at baseline was 65.9 (SD = 11.1) years. Lacunar stroke was the final diagnosis for 57% of the participants. 69% had hypertension, 22% had diabetes, 75% had hypercholesterolemia and 17% were current smokers or stopped smoking less than 1 year ago. Further, 9% had atrial fibrillation. The median NIHSS score was 1 (IQR 0–2), median mRS was 1 (IQR 0–1) and the mean MoCA score was 24.3 (SD = 3.6) at baseline visit. 

**Figure 2:**
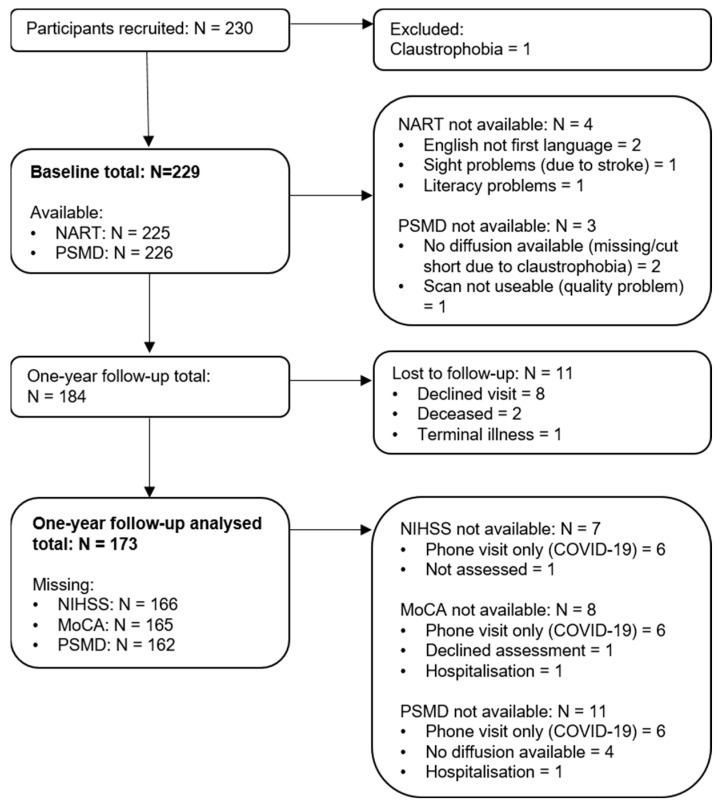
A flow diagram for data collection at baseline and 1-year follow-up. Note: At baseline N = 229 of which N = 226 had (useable) scans. At 1-year follow-up, N = 173 of which N = 162 had (useable scans). MoCA: Montreal Cognitive Assessment; NART: National Adult Reading Test; NIHSS: National Institute of Health Stroke Scale; PSMD: Peak width of Skeletonized Mean Diffusivity.

At the time of the current analysis, 173 (31.8% female) participants had attended their 1-year follow-up and are included in longitudinal analysis. Mean age at follow-up was 67.5 years (SD = 11.0). Out of the 184 participants who were eligible for the 1-year follow-up, eight participants declined the visit, two were deceased and one participant had a terminal illness and could not attend. For one other patient, who was hospitalized at the time of their 1-year visit and, therefore, could not attend, any available data were collected from hospital medical records. See Figure 2 for an overview of collected and missing data. As the study is still ongoing, the number of follow up scans is expected to increase for future analyses.

### 3.2. Cross-Sectional Associations between PSMD and Global Cognition at Baseline and 1-Year Visit

At baseline, we did not find associations between PSMD and the MoCA. Lower MoCA scores were associated with older age (standardized β = −0.309, 95% confidence interval [CI] −0.433 to −0.185) and higher NIHSS score (standardized β = −0.201, 95% CI −0.321 to −0.082) (Table 2). Higher MoCA scores were associated with higher NART correct scores (standardized β = 0.417, 95% CI 0.310 to 0.525). We did not find associations between sex, or mRS with the MoCA. 

At the time of the 1-year visit, there was an association between MoCA and PSMD (Table 3). Lower MoCA scores were associated with higher PSMD (standardized β = −0.301, 95% CI −0.434 to −0.168), higher NIHSS scores (standardized β = −0.220, 95% CI −0.357 to −0.084), and older age (standardized β = −0.218, 95% CI −0.350 to −0.085). Higher MoCA scores were still associated with higher NART scores (standardized β = 0.406, 95% CI 0.286 to 0.562).

### 3.3. Longitudinal Analysis of Baseline PSMD and 1-Year Global Cognition

Baseline predictors of 1-year MoCA scores were the baseline MoCA score (standardized β = 0.502, 95% CI 0.360 to 0.644), NART (standardized β = 0.144, 95% CI 0.017 to 0.271) and PSMD (standardized β = −0.182, 95% CI −0.308 to −0.056). Age, sex, baseline mRS and NIHSS scores were not associated with MoCA at 1-year visit, Table 4.

## 4. Discussion

We investigated associations between PSMD and post-stroke global cognition and examined whether these associations varied at different time-points. In the subacute phase, approximately two months after stroke, older age, lower premorbid IQ and worse stroke severity predicted worse global cognition, but we found no clear association between PSMD and MoCA. At one year after stroke, we did find a cross-sectional association between higher PSMD and worse global cognition. Our cross-sectional analysis at 1-year after stroke showed that PSMD is more strongly associated with global cognition than age and stroke severity. This is in line with our hypothesis. Interestingly, PSMD in the subacute phase is a possible predictor of global cognition at 1 year after stroke, as are baseline global cognition and premorbid IQ. 

Older age and (premorbid) IQ are well known predictors for post-stroke cognition and cognitive impairment. In general, older age and fewer years of education are related to lower scores on cognitive screening tests such as the MoCA [33]. Other studies that examined cognition after ischemic stroke or transient ischemic attack have also found that older age is a predictor of worse cognition and post-stroke cognitive impairment within months of stroke [25,44,45] and 1 year to 3 years post-stroke [2,4,25]. A similar study of participants with mild stroke [4] found that cognition at one year predicted cognition at 3 years after stroke, which is in line with our finding that cognition at three months predicted cognition one year after stroke. Premorbid IQ, and the NART as assessment of premorbid IQ, are predictors of post-stroke cognition [31,44,46,47]. However, age at baseline was not associated with 1-year cognition in our study, which is a novel finding. The effect of age might be confounded by pre-morbid intelligence, white matter structure as measured with DTI or stroke severity. Variables that reflect this, the NART, PSMD and NIHSS, are stronger predictors at 1-year after stroke. This is also supported by studies that show that low pre-morbid intelligence is related to worse post-stroke cognition [29], lower childhood IQ is related to higher SVD burden [48] and increased stroke risk [49], and that microstructure of white matter, as measured with DTI, supports general intelligence [50]. 

In line with previous studies, we also found an inverse association between post-stroke cognition and stroke severity, as measured with the NIHSS [1,2,51,52]. However, unlike previous studies, baseline stroke severity does not seem to predict cognition at one year after stroke in our data. 

We did not find any associations between sex and post-stroke cognition, which is consistent with a large meta-analysis of predictors of post-stroke dementia [53]. Some studies report that female sex is a risk factor for post-stroke cognitive impairment [2,10,54]. Any association between female sex and post-stroke cognition or stroke severity might be related to pre-stroke factors such as dementia, older age at time of stroke and pre-stroke dependency [53,55]. We recruited patients with lacunar ischemic stroke or similarly mild cortical ischemic stroke, and, therefore, are not able to assess the effect of the full range of stroke severities on post-stroke cognitive impairment. According to a population-based meta-analysis [55], women tend to have more severe ischemic strokes, NIHSS > 7, than men. We might have included fewer women due to our inclusion criterion of mRS ≤ 2 (slight disability) which might not reflect more severe strokes. However, a systematic review and meta-analysis assessing sex differences in SVD and people with ischemic stroke, which is similar to our mild stroke population, found that more men than women with SVD presented to hospital with ischemic stroke [56]. The inclusion criterion of mRS ≤ 2 impeded a large range of mRS scores to be included in these analyses, and this might also be an explanation for not finding any associations between mRS and post-stroke cognition at any of the time-points, while other studies do find associations between mRS and cognition [51]. However, a previous study in a similar population of mild stroke also did not find an association between mRS and global cognition at baseline and at 1-year follow-up [25], but they found an association between mRS and global cognition at three years. 

High values of PSMD, as an imaging marker of SVD, are thought to reflect worse white matter structure. The use of skeletonized MD maps for its calculation minimizes the effects of CSF contamination in MD measurements and PSMD has been shown to be more sensitive to changes in processing speed [12,19] and global cognition [21] than other diffusion-derived parameters. In our study, there is a hint in the direction of an association between PSMD and cognition at baseline, whereas at one year after stroke, the relationship is more defined. At one year, the standardized beta suggests that the effect of PSMD on cognition is larger than the effect of age and stroke severity, however, the effect is smaller than the effect of premorbid IQ as assessed by the NART. The results suggest that the relation between PSMD and global cognition at 1 year is stronger than within the subacute phase after stroke, and PSMD in the subacute phase also is of smaller predictive value to 1-year cognition than in the cross-sectional analysis at 1-year. It is not entirely clear why the relation between PSMD and cognition changes. For the longitudinal analysis, it might be explained by a smaller number of participants at 1-year, and, therefore, less power. It might reflect that the acute effects of stroke are ‘settling down’ after the subacute phase and that PSMD is a sensitive marker in predicting long-term outcomes. However, this needs further investigating, potentially in relation to changes in white matter. 

One of the strengths of our study is the large sample of longitudinal PSMD and cognitive data. Our study also includes valuable clinical data, and we correct for premorbid IQ in our analyses. To our knowledge, only two studies have longitudinal PSMD data available [20] or examined any longitudinal associations [18], in community-dwelling elderly and ischemic stroke patients respectively. Even fewer studies examined this PSMD data in relation to clinical outcomes, such as cognition [20]. Our additional strength is that our population consists of mild ischemic stroke patients, rather than community-dwelling elderly without diagnosed stroke. This gives us more insight in the relation between damage to the microstructure of white matter and cognition and the influence of acute clinical events. It is also important to study cognitive outcomes in a mild stroke population, since physical disabilities are less prominent than in severe populations and any cognitive problems might be overlooked. 

One of the limiting factors is the use of data from an ongoing study. While we were able to use all available baseline data, the 1-year follow-ups are still ongoing. This led to one year less data available than baseline data, with approximately 20% of the participants still to have their 1-year follow-up visit. This additional number of participants could possibly affect the cross-sectional 1-year and longitudinal analyses, but not the cross-sectional baseline analysis, and, therefore, we should be cautious with the interpretation of these preliminary results. Second, the inclusion of mild stroke patients means that the ranges of scores of the NIHSS and mRS are limited. We also used the mRS as a continuous variable in our analyses to avoid overfitting the models, while it might be more appropriately designated as an ordinal variable. The data were not amenable to analyses with an ordinal variable. Six participants only had phone visits at their 1-year follow-up visit. This was mainly due to the COVID-19 pandemic resulting in some participants being unable to attend in person due to restrictions, personal concerns regarding COVID-19, e.g., travel, attend visit in hospital, or they were deemed to have a high risk of complications from possible infection. Although efforts were made to gather as much information as possible via telephone interviews in these instances, some data, such as some aspects of cognitive assessments, the NIHSS and MRI could not be collected. It might be possible that people with complete follow-up data might be healthier than those who do not. However, since this is an ongoing study, we are not able to compare those groups as not all participants were yet eligible for their 1-year visits.

Future studies will benefit from larger, longitudinal datasets in stroke populations. This will make it possible to include more predictors in analyses. Potential predictors of interest would be vascular risk factors, imaging features of SVD and their change over time. It would also allow analyses to examine whether PSMD is an earlier and perhaps more useful predictor of cognition versus more overtly visible SVD features such as WMH. As outcomes, it would be interesting to look at more specific cognitive domains instead of global screening tests. Investigation of relations between PSMD and functional outcomes or disability require further longitudinal attention. A future research area might be whether different timeframes, e.g., acute phase versus subacute phase, show differences in PSMD. However, this would require frequent longitudinal imaging in the early stages after stroke or larger sample sizes to include participants with a larger range of time between stroke onset and baseline visit.

## 5. Conclusions

PSMD, a marker of SVD, sensitive to microstructural white matter changes, becomes more strongly associated with global cognition at 1 year after minor ischemic stroke, possibly once acute effects have settled. PSMD in the subacute phase after stroke might be able to predict long-term global cognition. Further studies are needed to examine underlying mechanisms of the differences of the influence of PSMD over time and potential relations to other clinical outcomes.

## Figures and Tables

**Table 1:** Demographic characteristics of participants at baseline and 1-year follow-up.

		N	Baseline	N	1 Year
Age, mean ± SD (range)		229	65.9 ± 11.1 (32.7–86.3)	173	67.5 ± 11.0 (33.7–87.4)
Sex, female (%)		229	77 (33.6)	173	55 (31.8)
Final diagnosis, lacunar stroke (%) *		229	130 (56.8)	173	94 (54.3)
Days between stroke and baseline visit, median IQR (range)		229	61, 43–76 (11–105)		
Hypertension, yes (%) *		229	157 (68.6)	173	116 (67.1)
Smoking, yes (%) *		229		173	
	Never		108 (47.2)		84 (48.6)
	Ex, >1 year		82 (35.8)		62 (35.8)
	Ex, <1 year		12 (5.2)		9 (5.2)
	Current		27 (11.8)		18 (10.4)
Diabetes, yes (%) *		229	50 (21.8)	173	38 (22.0)
Hypercholesterolaemia, yes (%) *		229	171 (74.7)	173	124 (71.7)
Atrial fibrillation, yes (%) *		229	21 (9.2)	173	15 (8.7)
NIHSS, median, IQR (range)		229	1, 0–2 (0–7)	166	1, 0–2 (0–14)
mRS, median, IQR (range)		229	1, 0–1 (0–2)	173	1, 0–1 (0–5)
MoCA, mean ± SD (range)		229	24.3 ± 3.6 (11–30)	165	25.0 ± 3.8 (10–30)
NART correct, mean ± SD (range) *		225	32.7 ± 9.6 (6–50)	170	32.2 ± 9.7 (7–48)
PSMD, mean ± SD (range), mm^2^/s × 10^−4^		226	0.238 ± 0.066 (0.141–0.595)	162	0.254 ± 0.086 (0.137–0.766)

* as recorded at baseline. IQR: Interquartile Range; MoCA: Montreal Cognitive Assessment; mRS: modified Rankin Scale; NART: National Adult Reading Test; NIHSS: National Institute of Health Stroke Scale; PSMD: Peak width of Skeletonized Mean Diffusivity.

**Table 2:** Associations with MoCA in cross-sectional linear regression at baseline.

	Standardized β	Standardized 95% CI	*p* Value
Age	−0.309	−0.433, −0.185	<0.001
Sex, male	0.031	−0.197, 0.259	0.790
NIHSS	−0.201	−0.321, −0.082	0.001
NART	0.417	0.310, 0.525	<0.001
mRS	−0.037	−0.152, 0.078	0.526
PSMD	−0.116	−0.241, 0.009	0.069

CI: Confidence Interval; mRS: modified Rankin Scale; NART: National Adult Reading Test; NIHSS: National Institute of Health Stroke Scale; PSMD: Peak width of Skeletonized Mean Diffusivity.

**Table 3:** Associations with MoCA in cross-sectional linear regression at 1-year follow up.

	Standardized β	Standardized 95% CI	*p* Value
Age	−0.218	−0.350, −0.085	0.001
Sex, male *	0.211	−0.054, 0.477	0.117
NIHSS	−0.220	−0.357, −0.084	0.002
NART *	0.406	0.286, 0.526	<0.001
mRS	−0.004	−0.142, 0.135	0.958
PSMD	−0.301	−0.434, −0.168	<0.001

* measured/reported at baseline. CI: Confidence Interval; mRS: modified Rankin Scale; NART: National Adult Reading Test; NIHSS: National Institute of Health Stroke Scale; PSMD: Peak width of Skeletonized Mean Diffusivity.

**Table 4:** Longitudinal linear regression baseline PSMD and 1-year MoCA.

	Standardized β	Standardized 95% CI	*p* Value
Age	−0.111	−0.242, 0.020	0.095
Sex, male	0.008	−0.232, 0.248	0.945
NIHSS	−0.058	−0.189, 0.072	0.379
Baseline MoCA	0.502	0.360, 0.644	<0.001
NART	0.144	0.017, 0.271	0.026
mRS	−0.064	−0.187, 0.059	0.304
PSMD	−0.182	−0.308, −0.056	0.005

All predictors are as measured/reported at baseline. CI: Confidence Interval; MoCA: Montreal Cognitive Assessment; mRS: modified Rankin Scale; NART: National Adult Reading Test; NIHSS: National Institute of Health Stroke Scale; PSMD: Peak width of Skeletonized Mean Diffusivity.

## Data Availability

The data reported are part of an ongoing study. Anonymized data will be made available upon reasonable request.
